# How the *FMR1* gene became relevant to female fertility and reproductive medicine

**DOI:** 10.3389/fgene.2014.00284

**Published:** 2014-08-29

**Authors:** Norbert Gleicher, Vitaly A. Kushnir, Andrea Weghofer, David H. Barad

**Affiliations:** ^1^Center for Human ReproductionNew York, NY, USA; ^2^Foundation for Reproductive MedicineNew York, NY, USA; ^3^Department of Obstetrics and Gynecology, Medical University ViennaVienna, Austria

**Keywords:** *FMR1 gene*, FSH, AMH, follicle maturation, fertility preservation

## Abstract

This manuscript describes the 6 year evolution of our center’s research into ovarian functions of the *FMR1* gene, which led to the identification of a new normal CGGn range of 26–34. This “new” normal range, in turn, led to definitions of different alleles (haplotypes) based on whether no, one or both alleles are within range. Specific alleles then were demonstrated to represent distinct ovarian aging patterns, suggesting an important *FMR1* function in follicle recruitment and ovarian depletion of follicles. So called *low* alleles, characterized by CGGn_<26_, appear associated with most significant negative effects on reproductive success. Those include occult primary ovarian insufficiency (OPOI), characterized by prematurely elevated follicle stimulating hormone (FSH) and prematurely low anti-Müllerian hormone, and significantly reduced clinical pregnancy rates in association with *in vitro* fertilization (IVF) in comparison to women with normal (*norm*) and *high* (CGGn_>34_) alleles. Because *low FMR1* alleles present in approximately 25% of all females, *FMR1* testing at young ages may offer an opportunity for earlier diagnosis of OPOI than current practice allows. Earlier diagnosis of OPOI, in turn, would give young women the options of reassessing their reproductive schedules and/or pursue fertility preservation via oocyte cryopreservation when most effective.

It has been known for decades that premutation range mutations of the fragile X mental retardation (*FMR1*) gene (CGGn_55-200_) are associated with greatly increased female risk of primary ovarian insufficiency (POI; Wittenberger et al., 2007). Neither endocrinologists nor geneticists, however, considered the possibility that the gene, beyond widely investigated neuro-psychiatric effects, giving it the name “fragile X chromosome,” may also have a role in ovarian function.

This idea arose at our center in 2008, and was immediately encouraged when we demonstrated that CGGn appeared in infertile women associated with follicle stimulating hormone (FSH) and anti-Müllerian hormone (AMH), both parameters of functional ovarian reserve (FOR; Gleicher et al., 2009a). Moreover, women with autoimmune-associated occult POI (OPOI, also called premature ovarian aging, POA) demonstrated significantly lower CGGn (in the new “normal” range) than non-autoimmune women with OPOI who, therefore, were presumed to have *FMR1*-related OPOI (Gleicher et al., 2009b).

The hypothesis of an *FMR1*-associated ovarian function effect was further supported when a literature review revealed a 1991 article by Fu et al. (1991) which, as a side note, in the general population reported a very prominent distribution peak at CGGn_30-31_ (**Figure 1**). It to us appeared “destined” to represent an additional function of the *FMR1* gene within what then was considered the normal range of CGGn. Seeing this spiking distribution peak in an otherwise spread-out distribution pattern, we suspected it to represent the gene’s hitherto unknown ovarian function.

**FIGURE 1:**
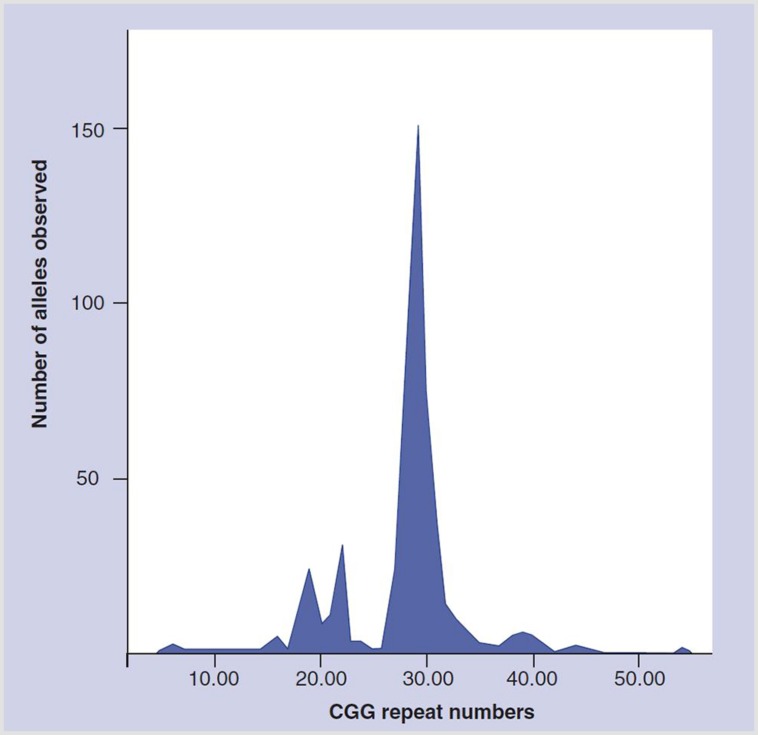
**Distribution of CGGn in general population.** Modified with permission from Fu et al. (1991).

We subsequently found a 2003 manuscript by Chen et al. (2003) reporting CGGn_30_ as the switching point between positive and negative message and maximal translation for the gene, which only further strengthened our conviction that we, likely, indeed had discovered a new ovarian function of the *FMR1* gene. It, however, remained to be determined what exactly this function entailed.

## DEFINING THE OVARIAN FUNCTION OF THE *FMR1* GENE

We then in a series of papers attempted to define how the *FMR1* gene affects ovarian function. First, we established the “normal” CGGn range for this presumed ovarian function of the gene at 26–34 (Gleicher et al., 2010c,d), a range that not only included at midpoint above noted switching point between positive and negative message at CGGn_30_ (Chen et al., 2003) but also the large distribution peak at CGGn_29-30_, reported by Fu et al. (1991; **Figure 1**).

Establishing a new “normal” range allowed for definition of new alleles (haplotypes) for the gene, which, of course, were distinctively different from the traditional CGGn mutations [normal, intermediate (“gray zone”), premutation and full mutation], used to define neuro-psychiatric risks, including the fragile X syndrome (Wittenberger et al., 2007). These new alleles (haplotypes) were also called genotypes, and were defined as normal (*norm*) if both alleles were in normal range, as heterozygous (*het*) if one allele was outside normal range and as homozygous (*hom*) if both alleles were outside normal range (Gleicher et al., 2010c,d).

Up to that point, the genetic literature, almost exclusively, had only concentrated on the expansive properties of the *FMR1* gene into abnormally high CGGn ranges. They were associated with well known neuro-psychiatric conditions at premutation (CGGn_∼55-200_) and full mutation range CGGn expansions (CGGn_>200_). Our center’s research, therefore, initially also concentrated only on expanding CGGn to the right of the by Fu et al. (1991) reported population peak at CGGn_29-30_ (Gleicher et al., 2009c,d). Because of the rather symmetrical distribution pattern of CGGn on both sides of the distribution peak (**Figure 1**), we, however, also initiated investigations of *low* alleles (CGGn_<26_), once we discovered that the risk for OPOI on both sides of the CGGn distribution peak was similar (Gleicher et al., 2009e).

As further discussed below, we quickly learned that *low* CGGn counts, indeed, appear associated with some of the most important effects of the *FMR1* gene on female reproduction. We, therefore, started subdividing above described “new” haplotypes (genotypes) into so-called sub-genotypes or *low* (CGGn_< 26_) and *high* (CGGn_>34_) alleles (Gleicher et al., 2010c,d).

Assessing young oocyte donors and infertile women at different ages, using these newly defined *FMR1* alleles, ovarian functions of the gene came into clearer view: different *FMR1* alleles were found associated with fairly typical ovarian aging patterns. Women with *norm* genotypes based on AMH levels followed a more or less normal ovarian aging pattern. *Het-low* carriers, in contrast, lost FOR at an accelerated pace, while carriers of *het-high* alleles throughout life appeared to recruit slower than other haplotypes and, therefore, at advanced ages present with best FOR (Gleicher and Barad, 2010; Gleicher et al., 2010c, 2012b,c).

Because *hom* patients are rare, especially when sub-divided into sub-genotypes, functions of *hom* haplotypes are not as well defined as *norm* and *het* patients. Preliminary, and mostly yet unpublished observations of *hom* populations suggest typical accelerated behavior if both alleles are either *high* or *low* but *high/low hom* patients appear to differ very significantly in ovarian phenotype, likely based on which allele is inactivated (Gleicher et al., unpublished data).

Based on these clinical observations we, therefore, concluded that the *FMR1* gene, likely, is involved in follicle recruitment, though in which way still, remains to be determined.

Additional observations added to the picture: while the normal range of CGGn_26-34_ is the same among all races, the distribution of individual alleles varies between races, with women of African descent demonstrating disproportionally more *low* and fewer *high* alleles, while Asian women (most were Chinese Han) demonstrate disproportionally more *high FMR1* and very few *low* alleles. Caucasians (including Hispanics), most diverse in *FMR1* allele distribution, fell in between the other two racial groups (Gleicher et al., 2010b, 2012a).

An increased prevalence of *low* alleles in women of African descent may, at least in part, contribute to their lower IVF pregnancy rates in comparison to Caucasian women, widely reported in the IVF literature (Gleicher et al., 2011). Women with *low FMR1* alleles, adjusted for covariates such as age, demonstrate significantly lower IVF pregnancy rates than women with *norm* genotypes (Gleicher et al., 2010d, 2011), an observation recently again confirmed in a greatly expanded study (Kushnir et al., 2014).

Though clinically often difficult to recognize, differences in ovarian aging patterns based on *FMR1* mutations can already be observed in young oocyte donors (Gleicher et al., 2013a) and, therefore, can also be utilized to optimize egg donor selection (Gleicher et al., 2010a). A 4 year follow up of young, carefully selected egg donors recently demonstrated that donors carrying *low FMR1* alleles already at their young ages significantly deviated from donors with *norm* and *high* alleles in FOR, as assessed by AMH levels (Kushnir et al., unpublished data).

Trying to better understand underlying physiological mechanisms for the observed IVF outcome differences between *FMR1* alleles, we discovered that women with *low* alleles convert dehydroepiandrosterone (DHEA) to testosterone (T) poorer than other mutations (Weghofer et al., 2012). Since LFOR is associated with low T levels (Gleicher et al., 2013b) and the increase in T after DHEA supplementation is predictive of pregnancy success in IVF (Gleicher et al., 2013c), this observation, at least partially, may explain lower IVF pregnancy rates in association with *low FMR1* alleles.

In over 5 years we, thus, succeeded in describing a well defined new function of the *FMR1* gene, which affects the ovarian aging process, reflected in FOR levels at different ages. Key studies by other investigators are briefly summarized below.

## CONCOMITANT OBSERVATIONS IN OTHER LABORATORIES

In a mouse model that carries a human *FMR1* premutation allele, Lu et al. (2012) reported that *FMR1* premutation RNA reduces the number of growing follicles in ovaries, and, thereby, impairs female fertility, possibly acting through the Akt/mTOR pathway. In another mouse model, a FX-PM mouse with 130 CGG repeats, Hoffman et al. (2012) demonstrated normal ovarian development and establishment of a normal primordial follicle pool. The animals, however, demonstrated much faster follicle loss, in concordance with what could be interpreted as the equivalent of OPOI (Hoffman et al., 2012). In an even more recent paper, Ascano et al. (2012) reported that a *Fmr1^-/-^* mouse model showed unexpected signs of premature follicular over-development. All of these animal studies, therefore, offer supporting evidence for a significant role of the *FMR1* gene in ovarian physiology.

Ferder et al. (2013) demonstrated in a rat model the expression of fragile X mental retardation protein (FMRP) in granulosa, theca and germ cells at all stages of follicle development. Moreover, these authors also demonstrated changes in *Fmr1* expression at protein as well as mRNA levels. Specifically, FMRP expression increased with advancing follicle development, with preantral and early antral follicles demonstrating similar *Fmr1* transcript, and decreased expression in preovulatory transcripts. The authors detected four different isoforms of FMRP during different stages of follicle maturation, with expression patterns varying from what they observed in brain and testis (Ferder et al., 2013).

Demonstration of similarly broad presence of *FMR1* at mRNA and protein levels in the human ovary would resolve further questions about the importance of this gene for ovarian physiology. Our center is currently conducting studies to further elucidate the relevance of *FMR1* mRNA and FMRP in different clinical situations affecting ovarian function.

Published clinical data from outside our laboratory have so far not included information on *low* CGGn alleles and, therefore, appear limited in scope and clinical relevance. Some, however, do offer interesting information: Schuettler et al. (2011), who assessed DNA and RNA samples from 74 women with idiopathic POI in order to evaluate the quantitative expression of *FMR1* in peripheral leukocytes and in relation to their CGGn is one example. Only women with POI demonstrated a large variance in *FMR1* transcript from leukocyte RNA samples, but this variance did not correlate to CGGn. They concluded that all women with CGGn_<26_ and/or CGGn_>34_, in other words with CGGn outside of the new “normal” range for ovarian function we had described, appear to experience what they called “relaxed” transcription control. As a consequence, they, therefore, are at risk for OPOI (i.e., premature ovarian aging).

Pastore et al. (2012) reported that women with LFOR present with overrepresentation of CGGn_35-44_ alleles, which in our mutation classification would mostly correspond to *high FMR1* alleles (Gleicher et al., 2010c,d). Similarly, Barasoain et al. (2013) reported in a Basque population with FSH levels above 10.0 mIU/mL, using traditional mutation designations for neuro-psychiatric risks, an increased prevalence of intermediate and premutation range alleles. These authors, therefore, also only commented on what we would describe as *high* allele patients.

But not all reported studied were able to demonstrate differences in CGGn between fertile and infertile women. De Geyter et al. (2014) were unable to demonstrate “expanded” CGGn in infertile women and Voorhuis et al. (2013), in our opinion incorrectly, dismissed a role for the *FMR1* gene in the ovarian aging process because CGGn was in their study not predictive of age at menopause.

While we never specifically investigated in our studies whether the *FMR1* gene affects age of menopause, we also noted that FOR curves for all the different *FMR1* alleles unite at advanced ages, suggesting a common menopause age for all of the haplotypes we have described. Different haplotypes, however, “take different roads” to that meeting point (Gleicher et al., unpublished data). We, therefore, do not agree with the conclusions reached by Voorhuis et al. (2013).

Furthermore, some reports do suggest a possible *FMR1* effect on menopause age: Sullivan et al. (2005) and Ennis et al. (2006) suggest such an effect likely around CGGn_<80_, a mid-range expansion size within the premutation range, concluding that menopause age is associated in a non-linear way with CGGn.

## THE FUTURE

Elucidating the function of *FMR1* mRNA and FMRP at different stages of follicle maturation appears of primary importance. Based on above described clinical observation we, indeed, would suspect that both may differ in association with different *FMR1* alleles (genotypes and sub-genotypes).

The *FMR1* gene may, however, also acquire additional significance as a prognostic diagnostic tool: we above noted that our data in young oocyte donors suggest that FOR in young women with *low FMR1* mutations already at very young ages declines significantly more rapidly than in women with either *norm* or *hom* alleles. Since approximately a quarter of all women carry a *low FMR1* allele (Gleicher et al., 2010c,d), this observation would suggest that approximately one quarter of the female population can be defined as “at risk” for LFOR at prematurely young ages.

If confirmed, assessments of *FMR1* mutations at young ages, possibly in association with other risk factors, could identify a high risk sub-population of young women, who, with careful longitudinal assessments of FOR, can be diagnosed at much younger ages than current practice allows for. Such early diagnosis of LFOR would then allow women to either enhance their reproductive timing or pursue fertility preservation at still young ages, when fertility preservation is most effective.

## Conflict of Interest Statement

David H. Barad and Norbert Gleicher are co-inventors of a number of pending patents, claiming diagnostic benefits from evaluations of CGGn on the *FMR1* gene.
